# Malaria knowledge and agricultural practices that promote mosquito breeding in two rural farming communities in Oyo State, Nigeria

**DOI:** 10.1186/1475-2875-9-91

**Published:** 2010-04-09

**Authors:** Oladimeji Oladepo, Grace O Tona, Frederick O Oshiname, Musibau A Titiloye

**Affiliations:** 1 Department of Health Promotion and Education, Faculty of Public Health, College of Medicine, University of Ibadan, Nigeria

## Abstract

**Background:**

Agricultural practices such as the use of irrigation during rice cultivation, the use of ponds for fish farming and the storage of water in tanks for livestock provide suitable breeding grounds for anthropophylic mosquitoes. The most common anthropophylic mosquito in Nigeria which causes much of the morbidity and mortality associated with malaria is the anopheles mosquito. Farmers are therefore at high risk of malaria - a disease which seriously impacts on agricultural productivity. Unfortunately information relating to agricultural practices and farmers' behavioural antecedent factors that could assist malaria programmers plan and implement interventions to reduce risk of infections among farmers is scanty. Farmers' knowledge about malaria and agricultural practices which favour the breeding of mosquitoes in Fashola and Soku, two rural farming communities in Oyo State were therefore assessed in two rural farming communities in Oyo State.

**Methods:**

This descriptive cross-sectional study involved the collection of data through the use of eight Focus Group Discussions (FGDs) and the interview of 403 randomly selected farmers using semi-structured questionnaires. These sets of information were supplemented with observations of agricultural practices made in 40 randomly selected farms. The FGD data were recorded on audio-tapes, transcribed and subjected to content analysis while the quantitative data were analyzed using descriptive and inferential statistics.

**Results:**

Most respondents in the two communities had low level of knowledge of malaria causation as only 12.4% stated that mosquito bite could transmit the disease. Less than half (46.7%) correctly mentioned the signs and symptoms of malaria as high body temperature, body pains, headache, body weakness and cold/fever. The reported main methods for preventing mosquito bites in the farming communities included removal of heaps of cassava tuber peelings (62.3%), bush burning/clearing (54.6%) and clearing of ditches (33.7%). The dumping of cassava tuber peelings which allows the collection of pools of water in the farms storage of peeled cassava tubers soaked in water in uncovered plastic containers, digging of trenches, irrigation of farms and the presence of fish ponds were the observed major agricultural practices that favoured mosquito breeding on the farms. A significant association was observed between respondents' knowledge about malaria and agricultural practices which promote mosquito breeding. Respondents' wealth quintile level was also seen to be associated with respondents' knowledge about malaria and agricultural practices which promote mosquito breeding.

**Conclusion:**

Farmers' knowledge of malaria causation and signs and symptoms was low, while agricultural practices which favour mosquito breeding in the farming communities were common. There is an urgent need to engage farmers in meaningful dialogue on malaria reduction initiatives including the modification of agricultural practices which favour mosquito breeding. Multiple intervention strategies are needed to tackle the factors related to malaria prevalence and mosquito abundance in the communities.

## Background

Malaria remains one of the world's worst health problems, as it leads to 1.5 to 2.7 million deaths annually [1-4]. These deaths primarily occur among children under five years of age and pregnant women in sub-Saharan Africa. These authors further reported that currently more people die from malaria than 30 years ago. In sub-Saharan Africa, malaria has been a major challenge to further improvement in child survival. This is especially so with malnutrition as a co-morbid condition coupled with delayed access to drugs during seasons of maximum transmissions [5,6].

In Nigeria, malaria impacts on development of the country as it causes death, reduces human work capacity or productivity in all sectors including the agricultural sector [7]. Malaria reduces Nigeria's GNP by 1.0% annually ($348 Million), and 25.0% of household income is expended on malaria control and treatment. Chloroquine, the cheapest drug for malaria treatment, has lost its potency, thus implying more cost for malaria control and reduction of income among agricultural workers [7]. According to the author malaria-afflicted farmers harvest only 40.0% of their crops compared with the healthy ones who could harvest almost 100.0% of their harvest. Malaria thus constitutes a great burden on the already depressed Nigerian economy through its severe impact on the agricultural sector.

Effective methods for reducing malaria infection exist but the challenge is their deployment in the context of weak health systems and the poverty situation in many parts of Africa, Asia and Southern America [7]. A recent effort has been directed at the use of anti-malarial drugs, and the promotion of insecticide treated bed nets (ITN) [7]. A new approach proposed by System-wide Initiative on Malaria and Agriculture (SIMA) which involves investment in malaria focused agricultural research has been reported [8]. The initiative entails providing health education to farmers, with the aim of reducing malaria at its source, in many high-risk regions. The approach involves management of land, water and farming practices in ways that discourage mosquitoes from breeding and to reduce human-mosquito contact. This is based on the fact that malaria is a disease that can be controlled by good management of the environment where the vector breeds.

The success of malaria control programmes relies heavily on community perceptions and practices related to the transmission, treatment and control of malaria [9]. These authors added that incorrect beliefs or inappropriate behaviour can interfere with the effectiveness of control measures, such as vector control and chemotherapy. This is particularly important in tropical areas where malaria control options are limited because of parasite resistance to anti-malarial drugs and the vector's resistance to insecticides. In such instances, an understanding of the communities' knowledge, perceptions and practices relating to malaria is crucial to the success of specific control measures.

The global management of malaria has passed through different stages over the years with differing goals. For instance, the global malaria management strategy moved from eradication and control in the 1950's and 60's to reduction of morbidity and mortality in the 1970's [10]. Much of the strategies employed from the 1980s till date involves early diagnosis and prompt treatment of affected persons. One of such efforts aimed at controlling morbidity and mortality is the programme geared towards early and appropriate management of childhood fevers in three communities in Nigeria [11].

Since the Roll Back Malaria (RBM) Initiative was launched in 1998, a new era has been ushered in which involves scaling up prevention and treatment activities and moving towards eradication [12]. There is need to examine the state of readiness of endemic farming communities regarding these initiatives. In Nigeria, the assessment of the knowledge and practices which promote mosquito breeding in agrarian communities has not been fully explored. Yet results from such study are useful for designing evidence-based malaria control interventions. This study was therefore designed to assess farmers' knowledge about malaria and to document their agricultural practices

## Methods

This study was carried out in two farming communities, Fashola and Soku in Oyo State, Nigeria. The communities were purposively chosen because they are centres of intensive traditional agricultural activities and some of their agricultural practices have potentials for promoting the breeding of the female anopheles mosquitoes. The decision to design the study to compare the knowledge and agricultural practices of farmers in Fasola and Soku was deliberate. Though the two communities are largely Yoruba settlements, they differ in terms of status and scale of agricultural activities. For instance, while Fasola has been a centre for livestock research since 1948, Soku is not. Though the Farmers in Fasola and Soku live and work in their farms with their families like most agrarian communities in Oyo State, the level of arable farming practiced in Fasola is higher than that of Soku. A comparative study approach was therefore adopted with a view to determining whether the factors which influence mosquito breeding in the two communities would be different or not. Each of the communities has an estimated population of 6,000, majority of whom are farmers cultivating food crops such as maize, cassava, yams, coco-yam, plantain, banana, tomato, okra, pepper and vegetables. Some farmers also engage in livestock and fish farming.

Most farms in Fasola and Soku are family owned farms. There is gender differentiation of farm-related roles in these communities, as is typical of most Yoruba agrarian settlements. Men are primarily responsible for the clearing, cultivation and weeding of farms while the major roles of women include harvesting, food processing and sale of agricultural produce. The average size of a farm in the study communities ranged from 1- 15 acres. Most farmers live in houses built with mud and roofed with leaves which are perceived to be having mosquito repellant properties. One Primary Health Care (PHC) center is located about two kilometers from Fasola and Soku.

It was decided to adopt combined use of Focus Group Discussion (FGD), Semi-structured interview and observation methods so that the strengths of one could effectively compensate for the weaknesses of the others. The choice of the FGD was based on the fact that it is more appropriate for documenting and describing social-cultural phenomena such as beliefs, perceptions and traditional practices. It was planned early enough to use the FGD results to facilitate the development of the semi-structured questionnaire and for noting physical phenomena which may need to be targeted for observation. The semi-structured interview was considered best for determining the frequency of beliefs, opinions, views, perceptions and reported practices relating to mosquito breeding and malaria upon which more valid generalizations could be based. The use of the observation method was considered more appropriate for the objective documentation of agricultural practices and other physical factors which can promote mosquito breeding. The design of the observation check-list was done after a review of literature relating to the breeding habits of mosquitoes. It was noted that the breeding habits of mosquitoes vary from species to species. For instance, while the *anopheles *mosquitoes breed in clear moving oxygenated water [10], *culex *mosquito prefer breeding in stagnant water bodies [12]. The *Aedes mosquitoes *breed in stagnant water in small containers in or around dwelling units [12]. Agricultural practices in Malaria endemic environments promote mosquito breeding [10] by creating opportunities for the presence of water bodies. It was therefore decided to design a checklist that could be used to document the presence of various kinds of water bodies including uncovered water-containing receptacles, trenches, dams, irrigation and food processing activities that cause pools of water to get collected in the farms.

Qualitative data were collected through eight FGDs with four conducted in each community, consisting of adult males aged 41 years and above, young males aged 20 to 40 years, adult females aged 41 years and above and young females aged 20 to 40 years. The FGD participants were purposively selected facilitated by use of the secretary to the Community Development Committee in each of the communities. During recruitment, participants were presented with an overview of the study including its objective and justification as well as venue and time for the FGDs. Only persons who provided oral informed consent were registered to participate in the FGDs. Each FGD group consisted of six to ten participants.

An FGD guide developed by the researchers was used to direct the FGD discussions moderated by one of the investigators with two research assistants (a male and a female) serving as note takers or recorders. The FGDs focused on the cause, dangers, perceived seriousness, treatment of malaria, areas where mosquitoes breed, efforts that farmers need to make to minimize mosquito breeding in the farm environment and suggestions on how health policies and laws could be used to promote environmental sanitation. Each FGD lasted between 35 and 45 minutes.

Quantitative data were collected with a pre-tested questionnaire and an observation checklist. The pre-tested semi-structured questionnaire was administered to a total of 403 respondents. The sample size was calculated using EPI-Info statistical package. Farms were scattered in different parts of each community. Consequently, stratified and simple random sampling technique was adopted. The investigator moved round the farms to interview respondents who consented to be involved in the study in each community. In the end, 199 respondents were interviewed in Fasola while 204 were studied in Soku. The respondents were interviewed individually. The FGD participants were excluded from being interviewed due to their prior exposure to information on issues being sought. Data were collected on respondents' demographic and socio-economic characteristics, cause, signs and symptoms of malaria and malaria prevention methods.

It is to be noted that the 403 respondents corresponded with 403 family farms (one male or female interviewee per farm). A random selection of 10.0% of the farms (40 farms) was scheduled for detail observation. Armed with an observation checklist, the investigators and research assistants moved round each farm and noted the presence or absence of agricultural practices as well as natural environmental conditions that could favour the breeding of mosquitoes. All the study instruments contained an informed consent note related to the voluntary nature of the study and steps taken to ensure the confidentiality of responses.

Informed consent was obtained at two levels- community and individual levels. At the community level, the opinion leaders were briefed about the nature and objectives of the study and the potential benefits associated with it. Communities were assured that information collected would not be used against their interests. Permission was granted before the study commenced in each community. Individual informed consent was obtained from interviewees and FGD discussants. The interviewees and discussants were provided with basic information about this study to enable them determine whether to participate or not.

The disclosed information included: study objectives and rationale, study design; potential risks (including lose of time) and how the risks would be addressed; societal benefits associated with participation; voluntary nature of participation; protection of participants' identity; and how confidentiality of responses would be maintained. Each respondent or focus group discussant was told that his/her names would not be written down on the instruments or on any other document and that information collected would not be shared with unauthorized persons. Owners of farms observed were briefed about the rationale behind observing their farm and the type of things that would be observed. They were however assured that information collected would not be shared with other farmers or any other person whatsoever. Data collection from interviewees focus group discussants and farms scheduled for observation did not start until after verbal informed consent had been secured. It was decided to use verbal informed consent approach because anecdotal records show that it was preferred to written informed consent in the study area.

The FGD data were transcribed from audio-tapes and subjected to content analysis based on categorized responses. A total of 403 questionnaires were administered (199 from Fashola and 204 from Soku) with deliberate efforts made to ensure gender representation. The questionnaire were sorted, cleaned, coded manually and entered into a computer. The data were analyzed using EPI-Info statistical software. Wealth-quintile ranges were calculated based on weighted frequency distribution of household items such as car, bicycle and radio owned by respondents. Respondents that scored 25.0% or less were categorized as belonging to low wealth-quintile group. The cut-off points for medium and high wealth-quintile were 50.0% and 75.0% respectively. Malaria knowledge score was calculated based on respondents' knowledge of the cause, signs and symptoms of malaria, anti-malaria drugs, malaria prevention, consequences of malaria illness, burden of malaria among farmers and agricultural practices which promote or prevent the breeding of mosquitoes.

## Results

### Focus Group Discussions

In all the FGD groups, only few participants stated that mosquito bite could lead to the occurrence of malaria. Many participants were of the view that malaria was caused by eating contaminated food, drinking of dirty water and staying long in the sun. Majority of the participants stated correctly that malaria could lead to death and that the disease was a serious illness that needs to be treated promptly.

The prevailing malaria treatment seeking behaviour in the two study communities were discussed and most FGD participants disclosed that use of local herbs was usually the first treatment action for malaria episodes. It was revealed further that use of local herbs could be followed by use of modern anti-malaria drugs such as chloroquine and fansider. Most FGD participants noted that people visit formal health care facilities such as clinics or hospital for care only when home management practices failed.

In response to a general question which focused on where mosquitoes breed, majority of the FGD participants stated correctly that mosquitoes could breed on the surface of stagnant water as well as on large leaves which hold water in bushy environments. It was suggested by discussants that in other to control or prevent mosquito breeding, farmers should always remove empty containers which can hold water and clear off stagnant water in the surroundings.

Another suggestion by most FGD participants which is not tied specifically to mosquito breeding but could also help in the control of mosquitoes' abundance was that the Nigerian government needs to enforce health policies and laws by deploying sanitary inspectors to communities to promote environmental sanitation

A question which focused on the cause of malaria in the community was broadly asked with a view to determining whether FGD participants would be able to link some of their agricultural practices to mosquito breeding and the prevalence of malaria. A clear difference in perception of the cause of malaria by chorological age was noted. Nine of the participants in both communities directly linked agricultural practices with mosquito breeding. A majority of the FGD participants aged 20 to 40 years irrespective of gender differences were however able to associate mosquito bites with the occurrence of malaria. The view that malaria is caused by working too hard in the heat of the sun cuts across both sexes among those aged 41 years and above with only few associating malaria occurrence with the bite of mosquitoes.

### Survey

The mean age of respondents was 38.7 ± 16.2 years. Majority (81.7%) of respondents were of the Yoruba ethnic group, 60.0% males, 67.2% married and 50.3% had secondary school education. Many (45.2%) of the farmers engaged in crop farming and were in the low wealth-quintile (48.1%). Nearly half (49.0%) obtained domestic water supply from rivers or streams (See table 1 for details of the socio-demographic characteristics).

**Table 1:** Demographic and socio-economic characteristics of respondents

Variables	Fasola (%)	Soku (%)	Total (%)
**Sex**			
Male	122(61.3)	120(58.8)	242 (60.0)
Female	77(38.7)	84(41.2)	161 (40.0)

**Marital status**			
Currently married	135 (67.8%)	136 (66.7%)	271(67.2)
Never married	56 (28.2%)	57 (27.9%)	113 (28.1)
Previously married	8 (4.0%)	11 (5.4%)	19 (4.7)

**Ethnic group**			
Yoruba	169 (84.9%)	156 (78.4%)	325 (81.7)
Hausa	16 (8.0%)	23 (11.5%)	39 (9.8)
Igbo	14 (7.1%)	20 (10.1%)	34 (8.0)

**Educational level**			
Never attended school (no formal)	39 (19.6%)	61 (29.9%)	100 (24.8)
Primary school	42 (21.1%)	32 (15.7%)	74 (18.4)
Secondary school	105 (52.8%)	98 (58.0%)	203 (50.3)
Higher Institution	13 (6.5%)	13 (6.4%)	26 (6.5)

*Type of farming engaged in			
Crop farming	128(61.2%)	69 (30.4%)	197 (45.2)
Food processing	58(27.8%)	72 (31.7%)	130 (29.8)
Mixed agriculture	6 (2.9%)	42 (18.5%)	48 (11.0)
Livestock farming	15 (7.2%)	24 (10.6%)	39 (8.9)
Fish farming	2 (0.9%)	20 (8.8%)	22 (5.1)

**Wealth--quintile**			
Low	77 (38.7%)	117 (57.4%)	194 (48.1)
Medium	74 (37.2%)	49 (24.0%)	123 (30.5)
High	48 (24.1%)	38 (18.6%)	86 (21.4)

*Source(s) of water supply for domestic use			
River/stream	141 (37.3%)	185 (64.5%)	326 (49.0)
Well	88 (23.3%)	72 (25.1%)	160 (24.1)
Bore hole	136 (36.0%)	25 (8.7%)	161 (24.2)
Well + tap	13 (3.4%)	5 (1.7%)	18 (2.7)

* Multiple answers allowed

#### Knowledge of Malaria

The semi-structured questionnaire used contained a 15-point knowledge scale for the assessment of the following malaria related knowledge issues: cure; signs and symptoms, prevention; consequences; treatment; and practices which promote and prevent the breeding of mosquitoes. Respondent's overall mean knowledge score was 9.1 ± 2.8 while the mean knowledge score for Fasola and Soku were 9.1 and 9.0 respectively with no significant differences when t-test was used to compare them.

Knowledge scores ranging from 0-5, 6-10 and 11-15 points were described as low, average and high respectively. Overall, majority (68.2%) had average knowledge scores. Few (6.8%) had low scores while 25.1% had high knowledge scores. These scores are indicative of the urgent challenges for health education in the study communities.

The respondents' perceived causes of malaria are shown in table 2. Most of the listed causes are misperceived. Consumption of contaminated food or water (35.4%) topped the list of such misconception followed by "staying for a long time in the sun" (20.0%). Only 12.4% could correctly link mosquito with the possible occurrence of malaria. Clearly, respondents' knowledge of the cause of the disease in the scientific par lance is abysmally low.

**Table 2:** Respondents knowledge of the cause of malaria

Cause of Malaria	Fasola community (N = 199)	Soku community (N = 204)	Total (%)
	
	Frequency (%)	Frequency (%)	Frequency (%)
Consumption of contaminated Food/water	147 (41.3)	84 (28.4)	231 (35.4)

Staying long in the sun	53 (14.9)	83 (28.1)	136 (20.0)

Dirty surroundings	54 (15.2)	38 (12.9)	92 (14.1)

*Mosquito bite	40 (11.2)	41 (13.9)	81 (12.4)

Stress	41 (11.5)	27 (9.2)	68 (10.4)

Rainy/cold weather	14 (3.9)	19 (6.5)	33 (5.1)

Body pains and headache	7 (2.0)	3 (1.0)	10 (1.6)

* Mosquito bite (most appropriate answer)

Few respondents could correctly outline the signs and symptoms used for recognizing a case of malaria (See table 3). The correctly listed possible signs and symptoms of malaria included high body temperature (13.8%); body pains (12.9%) headaches (8.3%), body weakness (7.7%) and cold (4.1%). The appropriate treatment seeking behaviour in terms of the time when treatment action should be initiated was sought. Most farmers in Fasola (95.0%) and Soku (89.7%) stated correctly that an episode of malaria should be treated promptly once signs/symptoms of the disease appear. Awareness of the preventable nature of malaria was also high among respondents in Fasola (88.9%) and Soku (86.7%). Farmers' relative vulnerability to malaria was determined. Overall, majority of the farmers in Fasola (95.0%) and Soku (87.2%) were of the perception that farmers are more susceptible to malaria compared with other people.

**Table 3:** Respondents' knowledge of the signs and symptoms of malaria

Signs and symptoms of malaria	Fasola community (N = 199)	Soku community (N = 204)	Total (%)
	
	Frequency (%)	Frequency (%)	Frequency (%)
Stomach pains	83 (23.2)	72 (24.3)	155 (23.7)

Yellow eyes	64 17.9	66 22.3	130 19.9

*High body temperature	61 (17.1)	29 (9.8)	90 (13.8)

*Body pains	43 (12.0)	41 (13.9)	84 (12.9)

*Head ache	31 (8.7)	23 (7.8)	54 (8.3)

*Body weakness	14 (3.9)	36 (12.2)	50 (7.7)

Yellow urine	32 (9.0)	14 (4.7)	46 (7.0)

* Cold/feverish	12 (3.4)	15 (5.0)	27 (4.1)

Constipation/diarrhea	8 (2.2)	-	8 (1.2)

Sleeplessness	5 (1.4)	-	5(0.8)

Cough	2 (0.6)	-	2 (0.3)

Mosquito bite	2 (0.6)	-	2 (0.3)

* Appropriate answers ** Multiple responses allowed

#### Practices related to Malaria prevention

The determination of respondents' practices for preventing mosquito breeding revealed that the clearing bushes around dwelling units with a view to eliminating hiding places for mosquitoes and removal of large leaves that can hold water were more practiced in Fasola (64.1%) than Soku (47.3%). Equal proportions of respondents in both communities (62.3%) removed heaps of cassava tuber peelings that can facilitate the collection of pools of water in the farms. A few respondents in Fasola (29.1%) and Soku (37.3%) cleared the ditches in their farms regularly with a view to interrupting mosquito breeding.

The methods used in homes to prevent mosquito bites are highlighted in table 4. The adoption of Insecticide Treated Nets (ITNs), a technology promoted through the Roll Back Malaria initiative in Nigeria, was low in both communities as only 6.1% of respondent in Fasola and 3.8% of their counterpart in Soku used ITNs. The other methods adopted by some respondents included use of the following: brooms for the physical killing of mosquitoes (25.3%), mosquito coils (25.9%); electric fan (18.7%); and insecticide sprays (11.9%). The other details are shown in the table 4.

**Table 4:** Methods respondents used for prevention of mosquito bites in their homes

Methods used for prevention of mosquito bites in homes	Fasola community (N = 199)	Soku community (N = 204)	Total (%)
	
	Frequency (%)	Frequency (%)	Frequency (%)
Killing of mosquitoes with broom	79 (21.9)	125 (28.0)	204 (25.3)

Mosquito coil	93 (25.8)	100 (22.3)	193 (23.9)

Electric fan	54 (15.0)	97 (21.7)	151 (18.7)

Insecticide sprays	63 (17.5)	33 (7.4)	96 (11.9)

Window/door screen	33 (9.2)	49 (11.0)	82 (10.2)

Insect repellant body cream	14 (3.9)	25 (5.6)	39 (4.8)

Insecticide treated bed-nets (ITN)	22 (6.1)	17 (3.8)	39 (4.8)

Mosquito candle	2 (0.6)	1 (0.2)	3 (0.4)

** Multiple responses allowed

### Observational data

The observed agricultural practices that favour mosquito breeding in the farms are depicted in table 5. Overall, the practice of dumping cassava tuber peeling was observed in 10 farms (50.0%) in each community. The practice of soaking peeled cassava tubers in water in large uncovered plastic containers was observed in 11 farms (55.0%) in Fasola and nine (45.0%) in Soku. The two aforementioned practices constituted the leading factors that promote mosquito breeding in the farms. Dug trenches were observed in 7 farms each in Fasola and Soku. The observed minor environmental factors with potential for promoting mosquito breeding was the presence of three dams in Fasola and one in Soku.

**Table 5:** Observed major agricultural practices that favour mosquito breeding on the farms

	Fasola community (N = 20)	Soku community (N = 20)	Total (%) (N = 40)
	
Observations	Frequency (%)	Frequency (%)	Frequency (%)
Dumped cassava peelings in the farm environment	10 (50.0)	10 (50.0)	20 (50.0)

Peeled cassava tubers soaked in plastic containers	11 (55.0)	9 (45.0)	20 (50.0)

Presence of dug trenches	7 (35.0)	7 (35.0)	14 (35.0)

Practice of irrigation	5 (25.0)	5 (25.0)	10 (25.0)

Presence of fish pond used for fish farming	3 (15.0)	3 (15.0)	6 (15.0)

** Only observed practices which promote mosquito breeding are displayed

Some other environmental conditions which favour mosquito breeding included presence of heaps of large dry leaves which hold water during the rainy season in both Fasola (65.5%) and Soku (40.0%), presence of run-off water from cassava fermentation spots in nine farms each in Fasola and Soku, uncleared stagnant water (Fasola, 35.0%; Soku, 55.0%), swampy environments (Fasola, 45.0%; Soku 30.0%) and presence of stored water for domestic use in uncovered tanks (Fasola, 20.0%; Soku, 55.0%).

## Discussion

Respondent's level of knowledge of the association between mosquito bite and malaria (12. 4%) is considered too low. It is not however very different from the results of a recent study conducted among five rural Yoruba communities in Ogun state, Nigeria which showed that only 11.7% attributed malaria to mosquito bite [11]. This development is not surprising. The linkage of malaria with the mosquito has no place in Yoruba indigenous knowledge and ethno-medicine. Studies among rural Yoruba populations often attribute malaria to a multiplicity of factors such as over-work, eating of too much palm oil, and working under the sun [13].

The signs and symptoms of malaria mentioned by respondents in the study are not different from those documented in other Nigeria studies [14-16]. In most malaria endemic communities, in South- western Nigeria, fever is the most frequently mentioned sign or symptom of malaria [14].

The low level of knowledge among the respondents relating to the causation or mode of transmission of malaria could, no doubt, foster the prevalence of malaria. This is so because since the incidence of malaria is not linked with the mosquito by most people, efforts will not be made to tackle the environmental health conditions and agricultural practices which promote the occurrence of malaria. The identified malaria knowledge deficit therefore poses a serious challenge that requires community-based health education intervention to address.

The farmers perceived malaria to be a very serious disease and that they were vulnerable to it. It was their view that the disease needs to be treated immediately its symptoms manifest. These are perceptions that promote positive health seeking behaviour in malaria disease [17-19].

The study shows that use of ITNs, a technology known to be highly effective in controlling mosquitoes, was not a common practice. Other researchers [13,20] have reported similar findings. The 2003 Nigeria Demographic and Health Survey (NDHS) [21] also revealed low ITN ownership in South-western Nigeria, a region which covers the area where this study was carried out. This is of concern because RBM has been promoting the use of ITN in the last 7 years. The lack of popularity of ITN may be due to its high cost, and scarcity in many rural communities.

In this study, a high proportion of respondents used relatively inexpensive methods such as the use of physical killing of mosquitoes and use of mosquito coils in preventing mosquito from biting them. The use of physical killing and mosquito coils is however characterized by some challenges. For instance, physical killing is laborious, limited in terms of the number of mosquitoes that can be killed and can only be adopted when the farmers are awake. The burning of mosquito coils on the other hand, does not kill mosquito; rather it is only effective in dazing and keeping mosquitoes at bay for a while. Mosquito become active and resume biting once they recover from the effects of the burning coils.

A study conducted in rural communities in Ebonyi state [17] has shown that bed nets are rarely used despite the abundance of mosquitoes. It is to be noted that generally use of bed net whether treated or not is not common in south western Nigeria. The results of the 2003 NDHS showed for instance that only 10.8% of households in the geographical zones of Nigeria owned any kind of net [18].

The farmers had good knowledge of how to minimize mosquito breeding in their farms. This is a welcome development. Advocacy and other behavioural change strategies such as social marketing and community development/organization need to be promoted among the farmers with a view to making them adopt sustainable use of appropriate environmental health technologies for controlling mosquito breeding.

The practice of good hygiene in homes and on farms is crucial to malaria control [19]. The author noted that such a practice was part of the measures which aided the eradication of malaria in the Netherlands in 1970s.

In order to control malaria effectively in Fasola and Soku some of the prevailing traditional food processing practices among farmers which favour the breeding of mosquitoes need to be addressed. The practices and environmental conditions of concern which need to be targeted for change or modification because they allow pools of water to collect in the farm include indiscriminate dumping of the peelings of cassava tubers, presence of heaps of leaves and run-off water from cassava fermentation sites.

Previous investigators have reported that cassava processing activities create opportunities for mosquitoes to breed. In Cameroon, it was observed that water holes burrowed for the retting of cassava tubers constitute breeding grounds for mosquitoes [20]. Cassava fermentation pools were major breeding sites for *culicine *mosquitoes in Nsukka, Nigeria [21]. It is necessary to note that the agricultural practices involving irrigation among farmers in this study are not bad in themselves; irrigation for instance promotes crop production. A major challenge associated with irrigation is that it creates a fertile ground for the breeding of mosquitoes. Educational interventions are needed to enhance the capacity of farmers to recognize the link between traditional food processing and irrigation activities and mosquito abundance.

The findings of this study relating to the link between agricultural practices and mosquito breeding are in agreement with the findings of some previous studies. For instance, it was noted in a study conducted in the farming district of South Arcot, India, that mosquito breeding in the district was promoted by some agricultural practices [22]. Generally use of small and large dams for agricultural purposes is known to favour the breeding of mosquitoes in sub-Saharan Africa [23].

The presence of stagnant water on rice farms and the collection of water in ditches and burrow pits constructed during agricultural practices have been reported to be conducive for malaria vector breeding [24]. In Nigeria, a study conducted in rural farming communities located in the North central parts of Ebonyi state [17] showed that rice farming promoted the breeding of *Anopheline *mosquitoes in swampy rice farms.

Multiple interventions are recommended for tackling the challenges identified in this study. The use of community organization and development is one of the interventions that hold great promise. It should entail enhancing the capacity of farming communities to be involved in community-directed interventions aimed at solving local problems as has been successfully done regarding the control of *Onchocerciasis *in rural communities in Oyo state. The critical step that will be involved in this approach include the following: identifying agricultural practices which promote mosquito breeding by communities themselves; identifying and selecting culturally appropriate mosquito control techniques; and the initiation of actions aimed at controlling mosquito abundance in the spirit of self-reliance and self-determination.

Community organization and development strategy has potential for ensuring sustainable community-based mosquito control initiatives in rural farm settlements. Though community- driven, the intervention needs to be facilitated by the agricultural extension and primary health care staff in the local government area.

Public enlightenment is another feasible intervention that can be adopted. The radio and traditional media such as the use of town criers which are common sources of information in most communities in Oyo state including the study area have crucial roles to play in this regard. Training is the third complementary intervention that needs to be conducted. It should be designed and implemented with a view to enabling farmers to acquire basic knowledge and skills relating to mosquito control, adoption of use of ITN and environmentally friendly food processing practices.

## Competing interests

The authors declare that they have no competing interests.

## Authors' contributions

OO conceived, designed and supervised the study. TGO participated in data collection and analysis and wrote the first draft. OF provided technical support to TGO during the collection and analysis of data. TM provided support during the design of the instrument and data collection, data analysis and interpretation. All the authors revised the drafts of this article until the final copy was produced.
